# Supplementary material for: Conditional chemoconnectomics (cCCTomics) as a strategy for efficient and conditional targeting of chemical transmission
Source: eLife. 2024 Apr 30;12:RP91927. doi: 10.7554/eLife.91927 (PMC11060718; doi:10.7554/eLife.91927)
Supplement: Figure 3—source data 3. [file elife-91927-fig3-data3.zip › Figure 3 source data3 part 2.zip!/Figure 3-Source data 3-part2/1053f3-1-CRISPResso_Report_t6djfp6u.zip!/CRISPResso_on_1053f3_2/1a.Read_barplot.pdf]

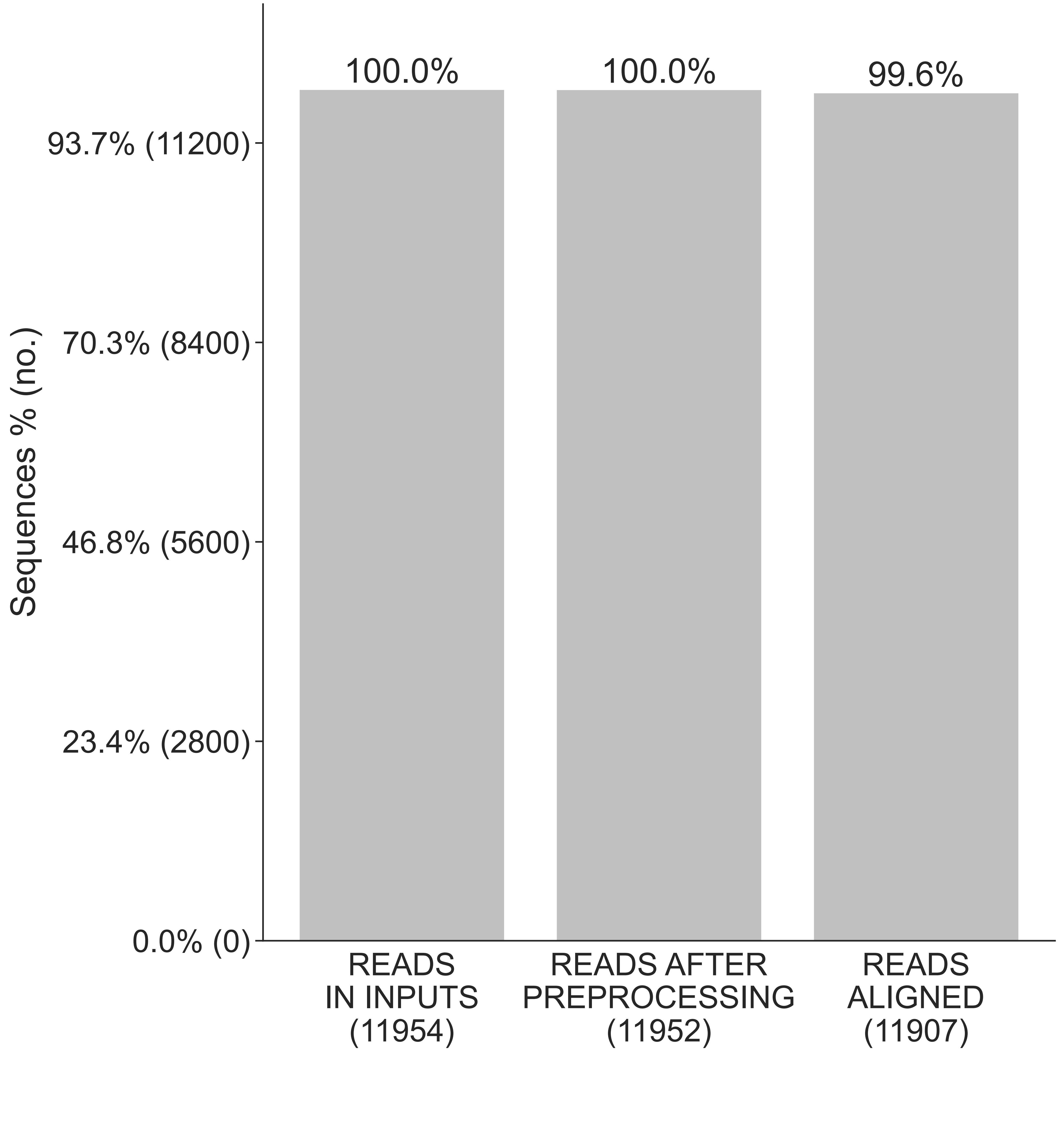

100.0%
100.0%
99.6%
93.7% (11200)
70.3% (8400)
46.8% (5600)
23.4% (2800)
0.0% (0)
Sequences % (no.)
READS IN INPUTS (11954)
READS AFTER PREPROCESSING (11952)
READS ALIGNED (11907)
